# A non-randomised single centre cohort study, comparing standard and modified bowel preparations, in adults with cystic fibrosis requiring colonoscopy

**DOI:** 10.1186/s12876-019-0979-z

**Published:** 2019-06-13

**Authors:** A. G. Matson, J. P. Bunting, A. Kaul, D. J. Smith, J. Stonestreet, K. Herd, R. S. Hodgson, S. C. Bell

**Affiliations:** 1 The Adult Cystic Fibrosis Centre. The Prince Charles Hospital. (TPCH), Queensland, Brisbane, Australia; 2 Department of Gastroenterology. TPCH, Queensland, Brisbane, Australia

**Keywords:** Bowel preparation, Colonic cleanse, Colonic lavage, Cystic fibrosis, Colonoscopy, Colorectal cancer screening, Gastrointestinal visualisation, Modified cystic fibrosis bowel preparation, Polyp detection, Colonoscopy screening

## Abstract

**Background:**

Adults with cystic fibrosis (CF) have been reported to be at five to ten-fold risk (25 to 30 fold risk after solid organ transplant) of colorectal cancer (CRC) than the general population. Limited publications to date have reported on practical aspects of achieving adequate colonic cleanse producing good visualisation. In this study, we compared two bowel preparation regimens, standard bowel preparation and a modified CF bowel preparation.

**Methods:**

A non-randomised study of adults with CF attending a single centre, requiring colonoscopy investigation were selected. Between 2001 and 2015, 485 adults with CF attended the clinic; 70 adults with CF had an initial colonoscopy procedure. After five exclusions, standard bowel preparation was prescribed for 27 patients, and modified CF bowel preparation for 38 patients. Demographic and clinical data were collected for all consenting patients.

**Results:**

There was a significant difference between modified CF bowel preparation group and standard bowel preparation group in bowel visualisation outcomes, with the modified CF bowel preparation group having a higher proportion of “excellent/good” GI visualisation cleanse (50.0% versus 25.9%) and lower rates of “poor” visualisation cleanse (10.5% versus 44.5%) than standard bowel preparation (*p* = 0.006). Rates of “fair” GI cleanse visualisation were similar between the two groups (39.4% versus 29.6%) (Additional file 1: Table S1). Detection rates of adenomatous polyps at initial colonoscopy was higher in modified CF bowel preparation cohort than with standard preparation group (50.0% versus 18.5%, *p* < 0.01). Positive adenomatous polyp detection rate in patient’s age > 40 years of age was higher (62.5%) than those < 40 years of age (24.3%) (*p* = 0.003). Colonic adenocarcinoma diagnosis was similar in both groups.

**Conclusion:**

This study primarily highlights that standard colonoscopy bowel preparation is often inadequate in patients with CF, and that colonic lavage using modified CF bowel preparation is required to obtain good colonic visualisation. A higher rate of polyps in patients over 40 years of age (versus less than 40 years) was evident. These results support adults with CF considered for colonoscopy screening at 40 years of age, or prior to this if symptomatic; which is earlier than CRC screening in the non-CF Australian population.

**Electronic supplementary material:**

The online version of this article (10.1186/s12876-019-0979-z) contains supplementary material, which is available to authorized users.

## Background

Cystic fibrosis (CF) affects more than 70,000 people globally, and adults outnumber children living with CF in many countries [1]. Cystic Fibrosis transmembrane conductance regulator (CFTR) protein dysfunction results in altered luminal electrolyte content in epithelial tissues and CFTR is present throughout all of the epithelia within the GI tract [2]. Chronic gastrointestinal (GI) symptoms in adults with CF are common, this may include distal intestinal obstruction, constipation, rectal prolapse, cholelithiasis, gastroesophageal reflux disease and GI cancer. Colorectal cancer (CRC) has been reported in patients with CF, and is even greater in those who have undergone transplantation (lung transplantation in particular being more prevalent in those with CF) [3–6].

The detection of CRC by symptoms in the general population, can lead to delayed diagnosis and many countries have programs to assist early detection [7, 8]. Australian population screening guidelines recommend faecal immunochemical tests (FIT) for adults from 50 years of age every one to two years; and those who have a positive test are advised to be referred for further investigation (e.g. colonoscopy) [7]. The diagnostic accuracy, speed and completeness of colonoscopy depend highly on the quality of the bowel cleansing preparation [9–11]. Even with established protocols, colonic stool clearance is incomplete in up to 25% of patients [12, 13].

Optimal CRC screening has not been established in patients with CF, although the CF CRC screening consensus recommendations 2018 have recently been published [6]. These recommendations suggest colonoscopy CRC screening in adults with CF, rather than alternative approaches (e.g. computed tomography colonography, stool-based tests, or flexible sigmoidoscopy). Non-invasive CRC screening approaches may be less reliable as swallowed occult haemoptysis and upper GI bleeding may lead to false positive testing. Colonoscopy has been proposed as the diagnostic modality of choice in patients with CF until further evidence is available [6], yet needs to be balanced against the increased anaesthetic risk, life expectancy, health status and quality of life. Technical factors may complicate effectiveness of colonoscopy including redundant colon (or excessive colonic length) and inadequate bowel preparation.

Over several years CF, transplant and gastroenterology services at The Prince Charles Hospital (TPCH), have observed a higher incidence of inadequate bowel cleanse with standard bowel preparation lavage regimens [14]. In this study, we compared two bowel preparation regimens: standard bowel preparation and a modified CF bowel preparation, on the effectiveness of colonic visualisation at time of colonoscopy. We also aimed to determine rates of adenomatous polyps and CRC in a screened CF population.

## Methods

A non-randomised cohort study of adults with CF, requiring colonoscopy investigation from TPCH; were selected. Between 2001 and 2015, 485 adults with CF attended our public government funded health system clinic. The state lung transplant service is also based at TPCH. The population included 70 adults with CF who had an initial colonoscopy procedure between 2001 and 2015. Five patients were excluded due to inability to determine the quality of the bowel preparation from the report or accompanying pictures. Standard bowel preparation was performed on 27 patients from 2001 to 2009, (data was collected retrospectively in this group after informed written consent obtained). A modified CF bowel preparation was prescribed after 2009 and informed written consent and all prospective data collected on 38 patients. Additional file 1: Table S1 summarises the characteristics of patients with cystic fibrosis at first colonoscopy procedure receiving standard bowel preparation (*n* = 27) and modified cystic fibrosis bowel preparation (*n* = 38).

Patients provided informed written consent for colonoscopy procedure for investigation of significant GI symptoms including rectal bleeding, constipation, and a family history of colonic cancer or as a part of lung transplant assessment procedures and written consent for data collection as part of this study. Patients requiring repeat surveillance colonoscopy procedures had data recorded on type of preparation and clearance reports. In four cases the initial colonoscopy was extended by one litre of GI lavage (due to inadequate bowel preparation) within 24 h of the initial procedure to permit a full colonoscopic investigation at the clinical discretion of the Gastroenterologist; such examinations within 24 h were counted as one initial colonoscopy procedure.

Colonoscopies performed prior to November 2009 included patients with CF who received standard bowel preparation (control group) with data collated retrospectively from medical records and gastroenterology database reports, written consent for data collection was obtained. The modified CF bowel preparation [Additional file 4] developed by CF Physicians, CF Dietitians and Gastroenterologists was introduced into practice from November 2009. For the purposes of this study from November 2009, consenting patient’s clinical details and adequacy of bowel preparation cleanse results was collected prospectively. Demographic and clinical data were collected on all consenting patients in the study including gender, age, post-lung transplantation, lung function, pancreatic insufficiency, CF-related diabetes, nutritional status, rates of adenomatous polyps and diagnosis rates of CRC. The modified CF bowel preparation protocol commenced 14 days prior to colonoscopy by the addition of an iso-osmotic laxative daily (the standard bowel preparation protocol does not include this laxative). Eight days prior to colonoscopy CF patients commenced a low fibre diet (compared to 4 days prior on standard bowel preparation protocol) and three days prior to colonoscopy CF preparation colonic purging was commenced, this included 1 sachet magnesium citrate in 250mls fluid, 3 Bisacodyl TM tablets, and 3 sachets of Glycoprep C TM in 3 l of fluid orally or via nasogastric / percutaneous endoscopic gastrostomy device and a clear fluid diet. Two days before colonoscopy, the CF bowel preparation included 3 sachets of Glycoprep C TM in 3 l of fluid/day spread over the day and a clear fluid diet (see Additional file 4) if there was a history of severe distal intestinal obstruction syndrome (DIOS), Gastrografin TM may have been also added by the CF Physician. One day before colonoscopy; 3 sachets of Glycoprep C TM in 3 l of fluid/day spread over the day and a clear fluid diet, again if there was a history of severe DIOS Gastrografin TM may have also been added by the CF Physician. On day of procedure a further 1 sachet of Glycoprep C TM in 1 l of clear fluid is added, no earlier than 4 h before scheduled colonoscopy (full details outlined in Additional file 4). The modified CF bowel preparation is longer in duration with up to 10 L of colonic lavage over 3–4 days compared to standard institutional bowel preparation (3-4 L colonic lavage 1–2 days prior to procedure) as detailed in Additional file 5).

The efficacy of the bowel preparation lavage was recorded using the Queensland Bowel Cancer Screening Program Bowel Preparation Descriptor scale [15] modified from the Boston Bowel Preparation Scale (BBPS) [16], which is applied to the Queensland CRC screening program. This scale grades cleansing adequacy of the entire colon, using descriptors excellent, good, fair or poor. The research group combined results for bowel cleanse resulting in “excellent” and “good” cleanse categories reporting as “excellent/good” cleanse outcomes due to the significance of reporting on these two categories of quality bowel preparation outcomes and small study numbers. All polyps removed were examined by two independent gastroenterological histopathologists. Number of colonic polyps, adenomatous polyps and detected colonic adenocarcinomas were recorded. The study received TPCH Human Research and Ethics Committee approval (HREC/12/QPCH/52).

### Statistical analysis

The primary outcome measure was efficacy of bowel preparation lavage documented on the colonoscopy report. Secondary outcome measures were the number of adenomatous polyps and colonic adenocarcinomas detected; duration of pre- and post-colonoscopy hospital length of stay. Student t-tests were used to compare continuous variables and Chi squared test (or Fishers exact test where appropriate) for categorical comparisons. A *p*-value of < 0.05 was considered significant. Data analysis was performed using PASW, version 18 (SPSS Inc., Chicago, IL, USA).

## Results

Baseline characteristics at first colonoscopy procedure were similar between the two groups (Additional file 1: Table S1). The proportion of subjects undergoing colonoscopy post-lung transplantation was similar between the two groups using standard (21.4%) and modified CF bowel preparation (24.3%).

There were significant differences between the modified CF and standard bowel preparation groups in colonic cleanse outcomes, with the modified CF bowel preparation group having a higher proportion of “excellent/good” GI cleanse compared to standard preparation (50.0% versus 25.9%) and lower rates of “poor” cleanse in the modified CF bowel preparation group than standard bowel preparation (10.5% versus 44.5%) (*p* = 0.006). Rates of “fair” GI cleanse were similar between the two groups (39.4% versus 29.6%) (Additional file 1: Table S1).

Detection rates of adenomatous polyps at initial colonoscopy was higher in modified CF bowel preparation cohort than with standard preparation (50% versus 18.5%, *p* = 0.01) (Additional file 1: Table S1).

Positive adenomatous polyp detection rate in patient’s age > 40 years of age was higher (62.5%) than those < 40 years of age (24.3%) (*p* = 0.003) (Additional file 2: Table S2). Colonic adenocarcinoma was diagnosed in two subjects in each group.

Repeat surveillance colonoscopes (20 procedures) in the CF Preparation cohort had similar rates of excellent/good bowel cleansing (Additional file 3: Table S3) during polyp surveillance intervals.

The median days hospitalised before the colonoscopy on a standard cystic fibrosis ward (standard preparation) was 2 days (25th–75th quartiles 0, 6 days) and the median days hospitalised after the colonoscopy was 1 day (25th -75th quartiles 0, 6 days). By comparison, the median days hospitalised before the colonoscopy (modified CF bowel preparation) was 3 days (25th–75th quartiles 0, 7 days) and the median days hospitalised after the colonoscopy was 1 day (25th -75th quartiles 0, 4 days).

There were no serious adverse events, e.g. deaths, colon perforations, ventilation or intensive care unit (ICU) admissions related to the colonoscopy procedure.

## Discussion

Increasing longevity of adults with CF reflects enhanced medical patient management and has led to greater numbers of adults with CF reaching middle age; with further growth expected over the coming decade [17]. The prevalence of emerging complications including GI malignancy has been reported [5, 6, 18, 19] with CRC reported to be five to ten fold higher risk compared to the general population, and 25 to 30 fold higher if the patient was post transplantation [6]. Survival rates for CRC in the general population significantly improve when detected and treated early; following the implementation of population-based screening programs in many countries and often based on faecal immunochemical testing (FIT) for people > 50 years of age [7]. However, lack of evidence for non-invasive CRC screening methods; in this high-risk group of patients has endorsed colonoscopic screening [6]. Positive colonoscopy screening relies on a high-quality CF bowel preparation due to challenges of the CF GI environment. The first CF CRC screening consensus recommendations [6]; suggest commencement of screening by colonoscopy at 40 years of age (30 years of age in organ transplant recipients) and 5 yearly re-screening or 3 yearly surveillance intervals. These recommendations are based on microsimulation screening analysis, based on balancing life years gained (LYG) and burden of screening within acceptable incremental cost-effectiveness ratios [6]. The CF Foundation (CFF) colonoscopy screening recommendations emphasise that these were developed for asymptomatic CF patients and stated that “physicians should recognize that CF is a colon cancer syndrome and consider diagnostic evaluation when patients present with new, suggestive symptoms or laboratory abnormalities” hence individual assessment may be required in patients less than 40 years of age [6].

The CFF [6] emphasise the importance of high-quality bowel preparation for optimal detection of colonic polyps especially as stool and GI mucoid physicochemical properties may complicate bowel preparation in patients with CF [6]. The CF GI environment is associated with GI dysmotility, defective chloride (and water secretion) into GI lumen, contributing to accumulation of viscid fecal material which may explain why the standard colon preparation protocol is less effective in achieving a thorough colonic lavage prior to colonoscopy.

In the general population, up to 25% of all colonoscopies are reported to have an inadequate bowel preparation [12, 13] the reported rate is < 25% at TPCH for all non CF patients (unpublished data). Inadequate bowel preparation has been correlated with lack of patient adherence with preparation instructions, medical conditions that make bowel cleansing more difficult, and unit-specific factors (e.g. delayed colonoscopy). Adverse consequences of ineffective bowel preparation include lower adenoma detection rates, longer procedure time, incomplete procedure, increased risk of electrocautery polyp complications, and shorter intervals between examinations [10, 13].

To date, limited publications have reported on practical aspects of colonic lavage in adults with CF. In our study, we report poor colonic lavage in 44% of patients with CF undergoing a standard colonoscopy preparation, yet this was reduced to 10% with the use of the longer modified CF bowel preparation. No difference was seen comparing efficacy of colonic lavage from initial and repeated surveillance procedures using the modified CF bowel preparation protocol. Research at another CF center has reported standard colonoscopy preparation protocol to be suboptimal and therefore lead to development of a CF-specific colonoscopy preparation in Minnesota USA [20]; although only 50% of patients underwent this preparation, and data were not reported as to adequacy of the CF-specific protocol when compared with standard colonic preparations. Details of standard bowel preparation (Additional file 5), modified CF bowel preparation (Additional file 4) and Minnesota protocols (Additional file 6) are provided.

Inadequate preparation in the general population has been associated with reduced adenoma detection rates [21]. Our study confirmed in those receiving modified CF bowel preparation, adenomatous polyp detection was significantly greater than a conventional approach to bowel preparation. The modified CF bowel preparation protocol developed at our center [Additional file 4], commences 14 days prior to colonoscopy by the addition of an iso-osmotic laxative daily. Eight days prior to colonoscopy CF patients commence a low fibre diet and three days prior to colonoscopy CF preparation colonic purging is commenced usually as an inpatient, linked to an admission on the public health care CF ward. Although such an intensive preparation may be more expensive in terms of additional GI lavage medication, and there may be a higher physical and mental cost with a longer duration of GI lavage & clear fluid diet than standard preparation, the advantages are of an adequate colonoscopy cleanse on the first attempt, minimizing the risk of missed pathology or the need for a repeat procedure with better preparation. Acceptance of a lengthier CF preparation may be improved if promoted by the healthcare team in terms of efficiency compared to standard preparation. In our centre the expertise of specialist CF Dietitians and Nurses is utilised to provide patient education on the modified CF bowel preparation protocol.

Our centre reported positive adenomatous polyp detection on initial scope rate of 50% (mean age 37.6 years) similar in incidence (49%) to the Minnesota CF cohort [22] (mean age 46.2 years). The Minnesota study [22] included patients greater than forty years old, in comparison to our slightly younger cohort. The current study, demonstrates a significant positive adenomatous polyp detection rate in patients forty years or older (62.5% v 24.3%) *p* = 0.003), supporting this earlier recommendation to initiate colonoscopy screening at age 40 for people with CF [6, 22]. Patients have been referred for colonoscopy to exclude colonic cancer prior to listing for lung transplantation without a formal age requirement to date, the mean age of patients undergoing colonoscopy has been 36.5 years.

### Study limitations

There are several limitations of this study that include our reporting of a single centre experience, which limited the population size. Secondly, the data collected included retrospective aspect of reporting effectiveness of colonoscopy preparation in the group prior to 2009, compared with prospective data collection after the introduction of the CF preparation protocol. Thirdly, the use of upgraded colonoscopy equipment during the study may have increased the precision of diagnosis of polyps in the latter cohort of patients. Finally we have reported no serious adverse events related to the colonoscopy procedure in patients receiving both standard and CF preparations, we did not collect formal data on less serious adverse events. Minor adverse events such as temporary weight loss, fluid overload and hypoglycaemia due to CF-related diabetes were noted in a few patients.

Colorectal cancer screening in a high risk cohort is an emerging issue in adult CF care centers’. The benefits and risks of such screening should be discussed in detail and needs to consider individual complexities including CF and non-CF comorbidities, life expectancy, safety, impact on health, treatments, and quality of life of the individual patient [6].

## Conclusion

This study primarily highlights that standard colonoscopy bowel preparation produces suboptimal colonic lavage and poorer colonic visualisation compared with a modified CF bowel preparation. A longer duration and larger volume of lavage using a modified CF bowel preparation (such as that developed by our CF Centre see Additional file 4) is required to obtain good colonic visualisation, in CRC screening or investigation. A higher rate of polyps in patients over 40 years of age (versus less than 40 years) was evident at our centre. These results support adults with CF considered for colonoscopy screening at 40 years of age, or prior to this if symptomatic; which is earlier than CRC screening in the non-CF Australian population.

### Further recommendations

Further research of other acceptable modified CF bowel preparations and / or less invasive screening methods should be prioritised, additionally assessment of cost benefit analyses of modified bowel preparations in this high-risk population may be of value to health care providers.

## Additional files

Additional file 1: **Table S1.** Characteristics of patients with CF at first colonoscopy procedure. (DOC 35 kb)

Additional file 2: **Table S2.** Comparison of age and rate of adenomatous polyp detection on first colonoscopy. (DOC 28 kb)

Additional file 3: **Table S3.** Comparison of initial and repeat scopes for efficacy of GI cleanse. (DOC 29 kb)

Additional file 4: The Adult Cystic Fibrosis Centre. The Prince Charles Hospital. (TPCH) Brisbane. The Adult Cystic Fibrosis Centre. The Prince Charles Hospital. (TPCH) Brisbane. Modified CF Bowel Preparation. (DOC 34 kb)

Additional file 5: Dept of Gastroenterology, The Prince Charles Hospital (TPCH) Brisbane. Preparation for Colonoscopy. STANDARD Bowel Preparation. (DOC 96 kb)

Additional file 6: Comparison of Bowel Preparations: 1-The Prince Charles Hospital (TPCH) Brisbane Australia Standard bowel preparation, 2- The Prince Charles Hospital (TPCH) Modified Cystic Fibrosis (CF) bowel preparation and 3 – University of Minnesota Colonoscopy Prep Instructions for patients with Cystic Fibrosis. (DOC 45 kb)

## Abbreviations

BBPS: Boston bowel preparation scale
CF: cystic fibrosis
CFTR: cystic fibrosis transmembrane conductance regulator
CRC: colorectal cancer
FIT: faecal immunochemical tests
GI: gastrointestinal

## Authors’ information

AM is a Senior Specialist Cystic Fibrosis (CF) Accredited Practising Dietitian (APD) and Credentialed Diabetes Educator (CDE). AM has worked in adult CF care for 20 years and has an interest in gastrointestinal CF complications.
