# Supplementary material for: A non-randomised single centre cohort study, comparing standard and modified bowel preparations, in adults with cystic fibrosis requiring colonoscopy
Source: BMC Gastroenterol. 2019 Jun 13;19:89. doi: 10.1186/s12876-019-0979-z (PMC6567575; doi:10.1186/s12876-019-0979-z)
Supplement: Supplementary file 1 — Table S1. Characteristics of patients with CF at first colonoscopy procedure. (DOC 35 kb) [file 12876_2019_979_MOESM1_ESM.doc]

**Additional file 1:**

**Table 1. Characteristics of patients with CF at first colonoscopy procedure.**

| **Patient characteristics** | **Standard Bowel Preparation**  **n=27** | **Modified CF Bowel Preparation**  **n= 38** | **p value** |
| --- | --- | --- | --- |
| Excellent & Good GI cleanse n (%)  Fair GI cleanse n (%)  Poor GI cleanse n (%) | 7 (25.9%)  8 (29.6%)  12 (44.4%) | 19 (50.0 %)  15 (39.4%)  4 (10.5%) | 0.006 |
| Sex female/male n (%) | 7/20  (25.9% / 74.0 %) | 18/20  (47.3% / 52.6%) | 0.12 |
| Mean age years (SD) | 35.0 (9.65) | 37.6 (9.58) | 0.57 |
| Post Lung transplantation (%) | 6 (21.4%) | 9 (24.3%) | 0.89 |
| Pancreatic Insufficiency n (%) | 25 (89.3%) | 35 (94.6%) | 0.72 |
| CFRD requiring insulin n (%) | 5 (17.9%) | 6 (16.2%) | 0.77 |
| Mean BMI kg/m^2^ (SD) | 22.0 (5.3) | 21.8 (4.3) | 0.54 |
| **Mean FEV_1_% predicted (SD) | 60.0% (14.3) | 49.1% (20.4) | 0.51 |
| Positive adenomatous polyp detection rate on initial colonoscope | 5 (18.5%) | 19 (50%) | 0.01 |
| Colonic cancer diagnosed n (%) | 2 (7.1%) | 2 (5.4%) | 0.72 |

Footnote: # excellent and good grading have been combined due to small patient numbers

**lung function in patients having colonoscopy pre-lung transplant only included: standard prep n=21, CF prep n= 29
