# Supplementary material for: A non-randomised single centre cohort study, comparing standard and modified bowel preparations, in adults with cystic fibrosis requiring colonoscopy
Source: BMC Gastroenterol. 2019 Jun 13;19:89. doi: 10.1186/s12876-019-0979-z (PMC6567575; doi:10.1186/s12876-019-0979-z)
Supplement: Supplementary file 2 — Table S2. Comparison of age and rate of adenomatous polyp detection on first colonoscopy. (DOC 28 kb) [file 12876_2019_979_MOESM2_ESM.doc]

**Additional file 2:**

**Table S2: Comparison of age and rate of adenomatous polyp detection on first colonoscopy.**

| **Polyp detection / Age** | **Age >40 years**  **n = 24** | **Age <40 years**  **n = 37** | **p value** |
| --- | --- | --- | --- |
| **Positive polyp detection rate** | 15 (62.5%) | 9 (24.3%) | 0.003 |
