# Supplementary material for: A non-randomised single centre cohort study, comparing standard and modified bowel preparations, in adults with cystic fibrosis requiring colonoscopy
Source: BMC Gastroenterol. 2019 Jun 13;19:89. doi: 10.1186/s12876-019-0979-z (PMC6567575; doi:10.1186/s12876-019-0979-z)
Supplement: Supplementary file 3 — Table S3. Comparison of initial and repeat scopes for efficacy of GI cleanse. (DOC 29 kb) [file 12876_2019_979_MOESM3_ESM.doc]

**Additional file 3:**

**Table S3: Comparison of initial and repeat scopes for efficacy of GI cleanse.**

|  | **Modified CF Initial Bowel Preparation**  **(n=38)** | **2+ repeat scopes Modified CF Bowel Preparation**  **(n=20)** | **p value** |
| --- | --- | --- | --- |
| **Excellent & Good GI Cleanse** | 19 (50.0%) | 12 (60.0%) | 0.53 |
| **Fair GI Cleanse** | 15 (39.5%) | 5 (25.0%) |
| **Poor GI Cleanse** | 4 (10.5%) | 3 (15.0 %) |
