# Supplementary material for: A non-randomised single centre cohort study, comparing standard and modified bowel preparations, in adults with cystic fibrosis requiring colonoscopy
Source: BMC Gastroenterol. 2019 Jun 13;19:89. doi: 10.1186/s12876-019-0979-z (PMC6567575; doi:10.1186/s12876-019-0979-z)
Supplement: Supplementary file 4 — The Adult Cystic Fibrosis Centre. The Prince Charles Hospital. (TPCH) Brisbane. The Adult Cystic Fibrosis Centre. The Prince Charles Hospital. (TPCH) Brisbane. Modified CF Bowel Preparation. (DOC 34 kb) [file 12876_2019_979_MOESM4_ESM.doc]

**Additional file 4.**

**Appendix 1:**

**The Adult Cystic Fibrosis Centre. The Prince Charles Hospital. (TPCH) Brisbane.**

**Preparation for Colonoscopy for adults with cystic fibrosis.**

**Modified CF Bowel Preparation.**

**Modified CF Bowel Preparation.**

- Day 14 before procedure commence 1 sachet Movicol ^TM^ /d.
- Day 8 before procedure commence low residue / low fibre diet (see below).
- Day 3 before procedure: 1 sachet magnesium citrate in 250mls fluid, 3 Bisacodyl ^TM^ tablets, and 3 sachets of Glycoprep C ^TM^ in 3 litres of fluid orally or via nasogastric / percutaneous endoscopic gastrostomy device and a clear fluid diet.
- Day 2 before colonoscopy; 3L Glycoprep C ^TM^ in 3 litres of fluid/day spread over the day and a clear fluid diet (see below), if there is a history of severe DIOS Gastrografin ^TM^ may also be added by the CF Physician.
- Day 1 before colonoscopy; 3L Glycoprep C ^TM^ in 3 litres of fluid/day spread over the day and a clear fluid diet (see below), if there is a history of severe DIOS Gastrografin ^TM^ may also be added by the CF Physician.
- On day of procedure a further 1 litre of Glycoprep C ^TM^ is added if procedure is in the afternoon, no earlier than 4 hours before scheduled colonoscopy.
