# Supplementary material for: A non-randomised single centre cohort study, comparing standard and modified bowel preparations, in adults with cystic fibrosis requiring colonoscopy
Source: BMC Gastroenterol. 2019 Jun 13;19:89. doi: 10.1186/s12876-019-0979-z (PMC6567575; doi:10.1186/s12876-019-0979-z)
Supplement: Supplementary file 5 — Dept of Gastroenterology, The Prince Charles Hospital (TPCH) Brisbane. Preparation for Colonoscopy. STANDARD Bowel Preparation. (DOC 96 kb) [file 12876_2019_979_MOESM5_ESM.doc]

**Additional file 5:**

**Appendix 2: Dept of Gastroenterology, The Prince Charles Hospital (TPCH) Brisbane.**

**Preparation for Colonoscopy. STANDARD Bowel Preparation.**

**STANDARD Bowel Preparation.**

- Day 2 to 4 prior to procedure commence low residue / low fibre diet (see below).
- Day 1 prior to procedure start bowel preparation (day 1 before procedure).
- Clear fluid diet (see below)
- 8am: magnesium citrate ^TM^ mix in 250ml hot water & refrigerate, also mix Glycoprep C ^TM^ in 3L water & refrigerate.
- 3pm drink magnesium citrate ^TM^ drink take 3 Bisacodyl ^TM^ tablets with 250ml water
- 5pm drink 2L of Glycoprep C ^TM^ @ 1litre per hour (finish by 730pm and diarrhoea should settle approx. 930pm, drink another 3 x 250mls clear fluids before bed.
- Day 0 / Day of procedure drink the 3^rd^ litre of Glycoprep C ^TM^ and clear fluids (see below), no earlier than 4 hours before scheduled colonoscopy.

***Low Residue / Low Fibre Diet:***

BREAD & CEREALS

| - Choose | - Avoid |
| --- | --- |
| - Plain white bread / toast | - Wholemeal, brown, rye breads |
| - Plain muffins | - Multigrain breads & muffins |
| - Plain bread rolls | - Wholegrain cereals, bran cereals, weetbix, vitabrits |
| - Crumpets, white bagels | - Cakes & biscuits containing nuts, dried fruit |
| - Plain Croissants | - Brown rice |
| - Pita bread | - Brown pasta |
| - Refined breakfast cereals: semolina, cornflakes, rice bubbles etc. | - Unprocessed bran, rolled oats, porridge, muesli |
| - Plain cakes & biscuits | - Brown crumpets |
| - Plain pancakes / pikelets | - Muffins with fruit |
| - White rice, white pasta, couscous | - Wholemeal flour and oatmeal |
| - Polenta |  |

FRUIT & VEGETABLES

| - Choose | - Avoid |
| --- | --- |
| - Potato without skin | - All other fruit & vegetables including salad vegetables |
| - Pumpkin without skin |
| - Marrow / Squash / Choko |
| - Avocado |
| - Mushrooms |
| - Ripe banana |
| - Apple, Peach with no skin |
| - Pears, Pawpaw, Rockmelon, Watermelon |
| - Canned peaches, apples, apricots & pears |

MEAT, EGGS, POUTRY, FISH & OTHER PROTEIN FOODS

| - Choose | - Avoid |
| --- | --- |
| - Beef | - Baked beans |
| - Pork | - Lentils, beans, chick peas |
| - Veal | - All legumes |
| - Lamb | - Casseroles |
| - Eggs | - Rissoles Stir fry meals |
| - Poultry | - Nuts & seeds |
| - Fish & Shellfish | - Pizza |

DAIRY FOODS

|  | - Avoid |
| --- | --- |
| - Choose | - Dairy products containing fruit, nuts or seeds |
| - Milk or powdered milk |
| - Ice cream |
| - Plain or vanilla yogurt |
| - Plain cheese |
| - Cottage cheese |
| - Custard |
| - Cream or sour cream |
| - Rice pudding |
| - Condensed milk, evaporated milk, |
| - Butter or margarine |

DRINKS

|  | - Avoid |
| --- | --- |
| - Choose | - Unstrained fruit juices, pulp juices - Red or green coloured drinks |
| - Water |
| - Tea |
| - Coffee |
| - Plain milk drinks |
| - Soft drinks |
| - Cordial |
| - Strained fruit juices |
| - Clear soups |
| - Milo or Ovaltine |
| - White wine |

OTHER FOODS

| - Choose | - Avoid |
| --- | --- |
| - Plain lollies | - Jams with skins / seeds |
| - Clear jellies ( | - Peanut paste / butter |
| - Sugar | - Dried fruit / nuts |
| - Plain or milk chocolate | - marmalade |
| - Honey | - Red jellies / lollies |
| - Jellied strained jams |  |
| - vegemite |

CLEAR FLUIDS / CLEAR FLUID DIET

| - Choose | - Avoid |
| --- | --- |
| - Clear or strained soup | - Any other fluids that you cannot see through |
| - Jelly (green or yellow) | - Avoid red & green coloured drinks |
| - Water or strained fruit juice |  |
| - Black tea / coffee / soft drink |
| - Herbal tea / iced tea |
| - Lemonade / Ginger ale / sports drink |
| - Herbal tea / iced tea |
| - Sports drink (yellow / orange) |
| - Cordial (yellow / orange) |
