# Supplementary material for: A non-randomised single centre cohort study, comparing standard and modified bowel preparations, in adults with cystic fibrosis requiring colonoscopy
Source: BMC Gastroenterol. 2019 Jun 13;19:89. doi: 10.1186/s12876-019-0979-z (PMC6567575; doi:10.1186/s12876-019-0979-z)
Supplement: Supplementary file 6 — Comparison of Bowel Preparations: 1-The Prince Charles Hospital (TPCH) Brisbane Australia Standard bowel preparation, 2- The Prince Charles Hospital (TPCH) Modified Cystic Fibrosis (CF) bowel preparation and 3 – University of Minnesota Colonoscopy Prep Instructions for patients with Cystic Fibrosis. (DOC 45 kb) [file 12876_2019_979_MOESM6_ESM.doc]

**Additional file 6:**

**Appendix 3: Comparison of Bowel Preparations: 1- The Prince Charles Hospital (TPCH) Brisbane Australia Standard bowel preparation, 2- The Prince Charles Hospital (TPCH) Modified Cystic Fibrosis (CF) bowel preparation and 3- University of Minnesota Colonoscopy Prep Instructions for patients with Cystic Fibrosis.**

| **Days prior to procedure** | **1. TPCH – Standard bowel preparation** | **2. TPCH - Modified CF bowel preparation 2** | **3. University of Minnesota CF preparation^20^** |
| --- | --- | --- | --- |
| 8-14 | n/a | Movicol 1 sachet/d | n/a |
| 7 | n/a | Low fibre / low residue diet | n/a |
| 6 | n/a | Low fibre / low residue diet | n/a |
| 5 | n/a |  | n/a |
| 4 | Low fibre / low residue diet | Low fibre / low residue diet | n/a |
| 3 | Low fibre / low residue diet | Clear fluid diet  Bowel preparation   - 1 sachet Magnesium citrate in 250mls fluid - 3 biscodyl tablets - 3L Glycoprep (spread over the day) | Low fibre / low residue diet |
| 2 | Low fibre / low residue diet | Clear fluid diet  Bowel preparation   - 3L Glycoprep (spread over the day) - Gastrograffin if history of DIOS | Low fibre / low residue diet  1L sports drink / hydration solution  Bowel preparation   - 4pm 1 sachet magnesium citrate in 295mls fluid. |
| 1 | Clear fluid diet  Bowel preparation   - 3pm 1 sachet Magnesium citrate in 250mls fluid - 3 biscodyl tablets - 5pm 2 L Glycoprep (1L /hour) | Clear fluid diet  Bowel preparation   - 3L Glycoprep (spread over the day) - Gastrograffin if history of DIOS | Low fibre / low residue diet until 1200 midday.  Bowel preparation   - 1000 3 biscodyl tablets   Commence Clear fluid diet after 1200 midday.   - 1500 - 1700 Commence GoLytely (2L): 8 x 240mls (240mls per 15 mins). - 2000 – 2200 Golytely (2L): 8 x 240mls (240mls per 15 mins). |
| 0 | Clear fluid diet  Bowel preparation   - 1L Glycoprep until 4 hours prior to colonoscopy | Clear fluid diet  Bowel preparation   - 1L Glycoprep until 4 hours prior to colonoscopy | 0700 Clear fluid diet & GoLytely (2L): 8 x 240mls (240mls per 15 mins).  Cease clear fluid diet 4 hours prior to colonoscopy. |
